# The Actin-Binding Protein Cortactin Promotes Sepsis Severity by Supporting Excessive Neutrophil Infiltration into the Lung

**DOI:** 10.3390/biomedicines10051019

**Published:** 2022-04-28

**Authors:** Nathaniel L. Lartey, Hilda Vargas-Robles, Idaira M. Guerrero-Fonseca, Alexander García-Ponce, Citlaltepetl Salinas-Lara, Klemens Rottner, Michael Schnoor

**Affiliations:** 1 Department of Molecular Biomedicine, Centro de Investigación y de Estudios Avanzados del Instituto Politécnico Nacional (CINVESTAV-IPN), Mexico City 07360, Mexico; smartlartey@gmail.com (N.L.L.); hildavargasr@yahoo.com.mx (H.V.-R.); idairaguerrerof@gmail.com (I.M.G.-F.); alexander.garcia@med.uni-muenchen.de (A.G.-P.); 2 Instituto Nacional de Neurología, Mexico City 14269, Mexico; cisala69@hotmail.com; 3 Division of Molecular Cell Biology, Zoological Institute, Technical University Braunschweig, 38106 Braunschweig, Germany; kle-rottn@tu-braunschweig.de; 4 Helmholtz Centre for Infection Research, Department of Cell Biology, 38124 Braunschweig, Germany

**Keywords:** sepsis, acute lung injury, cytokine storm, actin-binding protein, cortactin, neutrophil diapedesis, oxidative stress

## Abstract

Sepsis is a systemic infection that can lead to multi-organ failure. It is characterised by an uncontrolled immune response with massive neutrophil influx into peripheral organs. Neutrophil extravasation into tissues depends on actin remodeling and actin-binding proteins such as cortactin, which is expressed ubiquitously, except for neutrophils. Endothelial cortactin is necessary for proper regulation of neutrophil transendothelial migration and recruitment to sites of infection. We therefore hypothesised that cortactin plays a crucial role in sepsis development by regulating neutrophil trafficking. Using a murine model of sepsis induced by cecal ligation and puncture (CLP), we showed that cortactin-deficient (KO) mice survive better due to reduced lung injury. Histopathological analysis of lungs from septic KO mice revealed absence of oedema, reduced vascular congestion and mucus deposition, and better-preserved alveoli compared to septic wild-type (WT) mice. Additionally, sepsis-induced cytokine storm, excessive neutrophil infiltration into the lung and oxidative stress were significantly reduced in KO mice. Neutrophil depletion 12 h after sepsis improved survival in WT mice by averting lung injury, similar to both neutrophil-depleted and non-depleted KO mice. Our findings highlight a critical role of cortactin for lung neutrophil infiltration and sepsis severity.

## 1. Introduction

The worldwide incidence of sepsis in 2017 was 48.9 million people, with 11 million deaths [1]. In North America, 600,000 cases of sepsis are estimated to be recorded annually, with 30–50% mortality rate [2,3]. The Third International Consensus definition for sepsis (Sepsis-3) defines sepsis as a life-threatening organ dysfunction caused by a dysregulated and uncontrolled immune response to infection [4]. The mechanism of sepsis pathogenesis is multifactorial, but always involves increased vascular permeability and enhanced neutrophil recruitment [5]. Sepsis causes endothelial dysfunction due to aberrant activation of the endothelium by pro-inflammatory cytokines leading to increased expression of adhesion molecules and excessive transmigration of leukocytes into tissues [6].

Neutrophils are the first immune cells recruited in response to infection and inflammation [7]. They are well suited for combating pathogens as they can immediately release proteases and reactive oxygen species (ROS) [8]. While this rapid and aggressive response is beneficial during the immediate immune response to infection, systemic recruitment and activation of neutrophils during sepsis exacerbates their effector functions, thus contributing to host tissue damage and consequently organ failure [9,10,11]. Organ damage in sepsis has been linked to excessive neutrophil influx [10,11]. One of the first organs to fail in sepsis is the lung [12]. Sepsis causes Acute Respiratory Distress Syndrome (ARDS), and autopsy specimens from patients with multiple organ failure revealed excessive neutrophil infiltration into the lung [13].

Recruitment of neutrophils into tissues is a multistep process involving adhesive and migratory events such as tethering, rolling, arrest, crawling of neutrophils on the apical endothelial surface; and transendothelial migration [14]. The adhesive interactions between neutrophils and the vascular endothelium are in large part regulated by the actin cytoskeleton and actin-binding proteins (ABP) [5,14]. Cortactin is an ABP that associates with actin filaments and the Arp2/3 complex to control and stabilise actin branching [15]. Cortactin regulates many cellular processes including cell motility, endocytosis, assembly of intercellular junctions and host-pathogen interactions. Cortactin is ubiquitously expressed except for some haematopoietic cells including neutrophils [16]. Endothelial cortactin regulates vascular permeability, adhesion, and transmigration of neutrophils [17]. Importantly, cortactin-deficient mice are viable but have increased basal and induced vascular permeability [16]. It remains elusive why this basal vascular leakage does not cause an obvious phenotype. Surprisingly, increased permeability was accompanied by reduced neutrophil extravasation because of defective RhoG-mediated ICAM-1 clustering and neutrophil arrest on the vascular endothelium that prevented the neutrophils from exploiting the weakened endothelial cell contacts [16]. Therefore, we hypothesised that cortactin deficiency improves the outcome of sepsis by limiting the recruitment of neutrophils into the lung, thus preventing organ damage associated with excessive neutrophil recruitment.

## 2. Materials and Methods

### 2.1. Experimental Animals

Male C57BL/6J WT and cortactin KO mice (8–12 weeks old) were used for all studies. Mice were housed under specific pathogen-free conditions in the animal facility at CINVESTAV-IPN. Cortactin KO mice were generated by conditional gene ablation of exon 7 in the CTTN gene using the Cre-LoxP recombination method as described previously [16]. All experimental procedures were approved by the institutional animal care and use committee (IACUC) of CINVESTAV-IPN (protocol 0227-16; 08/12/16).

### 2.2. Induction of CLP (Cecal Ligation and Puncture) Sepsis

The CLP procedure was performed as previously described [18]. Briefly, mice were anaesthetised by intraperitoneal injection with ketamine (100 mg/kg body weight of mice) (Anesket, PISA, Mexico City, Mexico) and xylazine (10 mg/kg body weight of mice) (Procin, PISA, Mexico City, Mexico); then, the abdominal area was shaved and subsequently disinfected using 70% ethanol. A ~1.5 cm incision was then made in the linea alba, and the caecum was carefully exposed. A 4-0 sterile silk was used to ligate the caecum after which a 21 G needle was used to make a single puncture in the tip of the caecum, and 0.5 mm of faeces was exposed. The caecum was then returned to the peritoneal cavity, and the wound closed using a 4-0 sterile silk and disinfected. Finally, mice were subcutaneously injected with 1 mL of saline to induce the hyperdynamic phase of sepsis [18]. Sham-operated mice that underwent the same surgical procedure except for ligation and puncture served as controls. Animals were then returned to their cages with food and water provided ad libitum and monitored for 5 days for survival analysis.

### 2.3. Histology

Mice were sacrificed by anaesthesia overdose 24 h after CLP. For haematoxylin–eosin (H&E) staining, mice were perfused via the heart’s right ventricle with 20 mL of phosphate-buffered saline (PBS). The hepatic artery was then cut to allow fluids to drain. After tissue perfusion, the lungs were subsequently perfused via the trachea using 1 mL of 10% formalin until completely inflated and incubated for 5 min. Then, the lung was excised and submerged in formalin for 48–72 h. The formalin-fixed paraffin-embedded tissues were stained with H&E using standard protocols [19]. A semi-quantitative analysis of the lung was done in a blinded fashion by a pathologist who considered each of the following criteria and applied a histological score: presence of oedema, thickening of the alveolar wall, haemorrhage, vascular congestion, mucus deposition, and leukocyte infiltration. A score of 0 indicates no inflammation, while a score of 3 indicates severe lung inflammation and injury [20].

### 2.4. Haematology

Twenty-four hours after CLP/Sham surgeries, whole blood was recovered by cardiac puncture, transferred to ethylenediamine tetra-acetic acid (EDTA) tubes, and placed on a shaker for 20 min. Haematological analysis was then performed using the HemaVet automated haematology analyser (Drew Scientific, Dallas, TX, USA).

### 2.5. Plasma Cytokines

Peripheral blood obtained by cardiac puncture was treated with EDTA in Eppendorf tubes and placed on a shaker for 20 min. The plasma was obtained by centrifuging at 2500 rpm for 10 min. Plasma cytokines and chemokine (IL-6, IL-10, CCL2, TNF-α, IL-1β) were determined using the Milliplex 25plex-immunology Multiplex assay kit (Millipore, Burlington, MA, USA) per manufacturer’s instructions.

### 2.6. Quantitative Real-Time RT-PCR (qRT-PCR)

Total RNA was isolated using the Trizol (ThermoFisher Scientific, Waltham, MA, USA) reagent. RNAse-free DNase I was used to remove genomic DNA from the RNA preparation. Afterward, cDNA synthesis was done using the First Strand cDNA synthesis Kit (#K1612, Thermo Scientific, Waltham, MA, USA) according to the manufacturer’s instructions, and used for qRT-PCR. PCR was performed using a final volume of 10 μL containing 5.0 μL of Power SYBR Green PCR Master Mix 2X, 25 ng of cDNA from lung tissue homogenates, and 0.15 μM of both forward and reverse primer. The PCR was performed using the StepOne^TM^ Real-Time PCR System (Applied Biosystems, Waltham, MA, USA). PCR conditions: activation at 95 °C for 10 min, denaturation at 95 °C for 15 s. Denaturation was followed by annealing/extension at 60 °C for 60 s in 40 cycles. Following the last cycle, the melting curve was generated at 0.6 °C/s by heating from 60 °C to 95 °C. The gene used as an internal control of amplification was *Actb* (β-actin). The comparative expression of genes was quantified using the 2^−ΔΔCt^ method [21]. The following primer sets were used for the qRT-PCR. *β-actin:* Forward. 5′-TATCCACCTTCCAGCAGATGT-3′ Reverse: 5′-AGCTCAGTAACAGTCCGCCTA-3′. *IL-6:* Forward. 5′-CCTTCCTACCCCAATTTCCAA-3′ Reverse. 5′-AGATGAATTGGATGGTCTTGGTC-3′. *IL-1β:* Forward. 5′-GCAACTGTTCCTGAACTCAACT-3′ Reverse. 5′-TCTTTTGGGGTCCGTCAACT-3′. *IL-10:* Forward. 5′-ACTGCACCCACTTCCCAGT-3′ Reverse. 5′-ACTGCACCCACTTCCCAGT-3′. *TNF-α:* Forward. 5′-ACGGCATGGATCTCAAAGAC-3′ Reverse. 5′-AGATAGCAAATCGGCTGACG-3′. *CXCL-1:* Forward. 5′-TGTCAGTGCCTGCAGACCAT-3′ Reverse. 5′-CCTGAGGGCAACACCTTCA-3′. *CCL2:* Forward. 5′-CCCCAAGAAGGAATGGGTCC-3′ Reverse. 5′- GGTTGTGGAAAAGGTAGTGG-3′. *ICAM-1:* Forward. 5′-CAATTTCTCATGCCGCACAG-3′ Reverse. 5′-AGCTGGAAGATCGAAAGTCCG-3′. *VCAM-1:* Forward. 5′-TGAACCCAAACAGAGGCAGAGT-3′. Reverse. 5′-GGTATCCCATCACTTGAGCAGG-3′. *P-selectin:* Forward. 5′-TCATCCCGGTGAAGCAATGT-3′. Reverse. 5′-TGGAGAACGCAAGGACAGGTAT-3′.

### 2.7. Leukocyte Recruitment into the Lung

After sacrificing mice by anaesthesia overdose, lungs were perfused with 1× PBS until they turned white. The left lung was removed, put into 1.5 mL microtubes, and minced using fine scissors in 1 mL of 37 °C pre-warmed 1× PBS containing calcium and magnesium (0.9 mM) and 200 units of collagenase I (Sigma, St. Louis, MO, USA, C9891). Using a 21 G needle, a tiny hole was punctured into each tube cap to prevent the solution from becoming hypoxic. Then, the lung tissues were incubated at 37 °C in a water bath for 1 h. Cell suspensions were filtered using a 40 μm cell strainer to remove clumps and subsequently washed using 5 mL of 1× PBS, and centrifuged for 5 min at 180× *g*. The cell pellets were finally resuspended in 1 mL 1× PBS and used for flow cytometry staining and analysis.

### 2.8. Peritoneal and Bronchoalveolar Lavage

Twenty-four h after CLP, mice were euthanised by anaesthesia overdose and their abdominal surface sprayed with 70% ethanol. A total of 10 mL (two times 5 mL each) of cold PBS was injected into the peritoneum, and the peritoneal lavage fluid was recovered.

Bronchoalveolar lavage was performed as previously described [22]. Briefly, under anaesthesia, an incision was made in the mid-abdomen on the ventral side, and the skin and the muscle were removed to expose the thoracic cage, neck and peritoneal cavity. The thoracic cavity was opened to expose the lung and heart by cutting the diaphragm. The mice were perfused transcardially with 1× PBS. After tissue perfusion, the trachea was opened, and a small incision was made to allow the syringe with a 27-g needle to enter. A knot was made with silk to tie the needle to the trachea. Subsequently, 0.8 mL of PBS was slowly injected and aspirated 4 times. This was repeated for 3 more times (0.8 mL × 4). The fluid recovered was placed on ice, stained and analysed by flow cytometry.

### 2.9. Flow Cytometry

Peripheral blood cells, peritoneal lavage fluid (PLF), bronchoalveolar lavage fluid (BALF), and lung tissue cell suspension were recovered as described above. Red blood cell lysis in whole blood was done using BD Pharm Lyse^TM^ (BD Biosciences, San Jose, CA, USA) lysing buffer. Cells in all samples were counted using a Neubauer chamber, and an equal number of 500,000 cells were blocked with TruStain FcX anti/mouse CD16/32 (BioLegend, San Jose, CA, USA) for 20 min at room temperature in PBS containing 3% FBS. Neutrophils were detected with an antibody against Ly6G (APC-Cy7-coupled 1:100, BioLegend, San Jose, CA, USA, clone 1A8) and total leukocytes with an antibody against CD45 (Pacific blue-coupled 1:100, BioLegend, clone 30-F11). All data acquisition was performed on a BD LSR Fortessa (BD Biosciences, San Jose, CA, USA). Data were analysed using FlowJo Treestar V10 software (FlowJo, Ashland, OR, USA).

### 2.10. Bacterial Counts

The whole blood and peritoneal lavage fluid were serially diluted with 0.9% saline and bacterial colony-forming units (CFU) determined by inoculating serially diluted samples on tryptic soy agar plates (MCD Laboratory, Mexico-City, Mexico), followed by incubating them at 37 °C for 24 h. CFU were then counted.

### 2.11. Oxidative Stress Assay

Twenty-four h after CLP, mice were anaesthetised and transcardially perfused with 20 mL PBS until the lungs turned white. Afterward, the lung was perfused with Tissue-Tek and subsequently harvested. Cryosections of 8 µm thickness were cut and placed on a glass slide. Dihydroethidium (DHE) (Life Technologies, Grand Island, NY, USA) was re-suspended in dimethyl sulfoxide (DMSO) to obtain a stock concentration of 10 mM. 500 μL of DHE solution was then topically applied to each tissue section, and mounted using a coverslip. Slides were incubated in a dark, humidified incubator at 37 °C for 30 min. DHE was counterstained with DAPI (4,6-diamidino-2-phenylindole dihydrochloride). Sections were examined within 8 h after the incubation period using a confocal laser scanning microscope (Leica TCS, SPE, Leica Microsystems, Wetzlar, Germany) and images taken using a 40× objective.

### 2.12. Neutrophil Depletion

Neutrophil depletion was performed as previously described [10]. Briefly, mice were intravenously injected with 25 μg of monoclonal anti-mouse Ly6G/Ly6C (Gr-1) antibody 12 h after CLP surgeries. Control mice were injected with rat IgG2b, κ isotype control antibody at the same concentration. The mice were subsequently returned to their cages and monitored for about 30 min. Twenty-four hours after CLP sepsis (12 h after the injection of the antibodies), the animals were euthanised by anaesthesia overdose, and blood samples, PLF, BALF, and lung tissues recovered as described above.

### 2.13. Statistical Analysis

Data were analysed using GraphPad Prism version 6.0 (GraphPad Software, San Diego, CA, USA). All results were expressed as means ± standard error of the mean (SEM). The differences among groups were determined using a one-way analysis of variance (ANOVA) followed by Tukey’s post hoc analysis. Differences between 2 groups were analysed by unpaired *t*-test. *p*-values < 0.05 were considered statistically significant.

## 3. Results

### 3.1. Cortactin Deficiency Improves Survival and Protects against Sepsis-Induced Lung Damage

**Figure 1:**
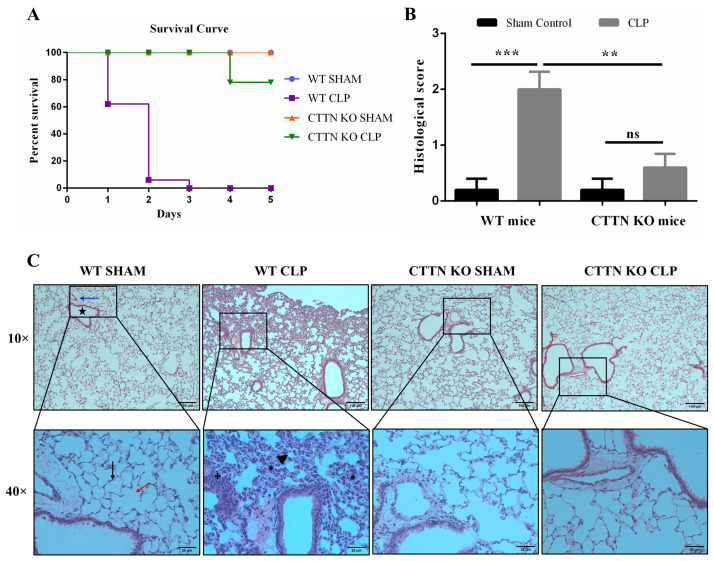
Cortactin gene ablation improves survival and protects against sepsis-induced lung tissue damage. (**A**) Five-day survival curve of mice subjected to CLP or Sham surgeries. Animals monitored for 5 days for survival. n = 10 for WT/CTTN KO Sham, n = 10–12 for WT/CTTN KO CLP. (**B**) The histological score of inflammatory changes in the lung tissue using the following parameters: alveoli space closing and collapse, alveolar wall thickening and fibrosis, oedema, haemorrhage, and leukocyte infiltration. 0: absence of inflammation, 1: low inflammation, 2: moderate inflammation, 3: high inflammation. (**C**) Representative histological images of lung tissue cross-sections. Sham-operated WT and CTTN KO mice showed normal lung tissue histology characterised by a normal alveolar area (red arrow), single cell layer thick alveolar wall (black arrow), normal bronchiolar area (star), preserved blood vessels (blue arrow), absence of leukocyte infiltration, and oedema. WT CLP mice showed major signs of lung inflammation and injury seen as massive leukocyte infiltration (+), alveolar wall thickening (asterisks), and alveolar space closing and collapse (black arrowhead). Data are represented as mean ± standard error of the mean (SEM) of at least 5 animals. ** *p* < 0.01, *** *p* < 0.001, ns: non-significant. CTTN: cortactin; CLP: Cecal ligation and puncture.

Sepsis and sepsis-associated multi-organ failure are characterised by the excessive influx of neutrophils into peripheral organs such as the lung. Due to the role of cortactin in supporting ICAM-1 functions to promote neutrophil extravasation, we hypothesised that cortactin promotes sepsis severity by supporting neutrophil influx into organs. To test this hypothesis, the effect of cortactin deficiency on sepsis induced by CLP was investigated. Mice subjected to sham surgeries had 100% survival at the end of the 5-day study period (Figure 1A). Septic WT mice had 40%, 95% and 100% mortality at day 1, 2 and 3, respectively. Septic CTTN KO mice showed an improved survival with only 25% of mice dying over the 5-day experimental period. Thus, cortactin deficiency exerts a protective effect on mice subjected to CLP sepsis.

Having observed that septic CTTN KO mice had better survival than their septic WT littermates, the underlying mechanisms regulating this effect was investigated. Acute lung injury (ALI) has been linked to deaths during sepsis; and the lung is among the first organs to fail during sepsis [23]. Thus, histopathological analyses of H&E-stained lung tissues from sham-control and septic WT and CTTN KO mice were performed to assess the degree of ALI. Sham-operated WT and CTTN KO mice had normal lung histology evidenced by one-cell thick alveoli wall, normal alveolar and bronchiolar area, absence of oedema and mucus deposition, non-congested blood vessels and no infiltration with leukocytes (Figure 1B,C). As expected, lung tissues of septic WT mice showed significant signs of inflammation and injury, namely thickening of the alveolar walls, shrinkage and collapse of the alveoli, mucus deposition and fibrosis, and excessive infiltration with leukocytes. In contrast, CTTN KO mice subjected to CLP sepsis showed a much better preserved lung tissue histology similar to the sham controls (Figure 1B,C). Histological scoring revealed a significantly higher score for septic WT mice (2.0 ± 0.3) compared to WT sham controls (0.2 ± 0.2) and septic CTTN KO mice (0.6 ± 0.3) (Figure 1B). Hence, the improved survival of septic CTTN KO mice is likely the result of less lung damage.

### 3.2. Cortactin Deficiency Attenuates the Inflammatory Response in the Lung during Sepsis

**Figure 2:**
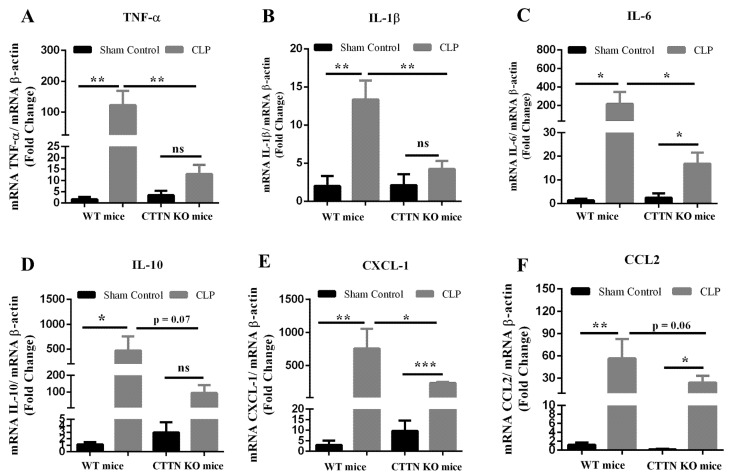
Cortactin deficiency attenuates the inflammatory response in the lung during sepsis. Relative gene expression of the pro-inflammatory cytokines (**A**) TNF-α, (**B**) IL-1β, (**C**) IL-6, (**D**) IL-10, and chemokines (**E**) CXCL-1, and (**F**) CCL2 was determined in the lung tissue 24 h after CLP or Sham surgeries. The ΔΔCt method was used to determine differences in gene expression shown as fold change normalised to the housekeeping gene β-actin. Data are represented as mean ± SEM of at least 5 animals per group. * *p* < 0.05, ** *p* < 0.01, *** *p* < 0.001, ns: non-significant.

Given the reduced signs of inflammation and lesser lung injury in septic CTTN KO mice, the mRNA levels of inflammatory mediators in the lungs after sepsis were determined using quantitative real-time RT-PCR. CLP sepsis caused increased expression of the pro-inflammatory cytokines TNF-α, IL-1β, IL-6, as expected (Figure 2A–C). The lungs of septic CTTN KO mice showed a significantly reduced pro-inflammatory cytokine response compared to septic WT mice (Figure 2A–C). CLP in WT mice also significantly increased the expression of the anti-inflammatory cytokine IL-10 (471.9 ± 284.4); and septic CTTN KO mice showed a tendency towards lower levels of IL-10 mRNA albeit not in a statistically significant fashion (94.13 ± 48.2) (Figure 2D).

CXCL-1 and CCL2 are chemokines crucial for the recruitment of neutrophils and monocytes during inflammation; and sepsis induces chemokine receptor expression on peripheral neutrophils [24]. As expected, CLP sepsis in WT mice caused significantly increased expression of both CXCL-1 and CCL2 compared to sham control. Notably, the lungs of septic CTTN KO mice showed reduced expression of these chemokines (Figure 2E,F), which was statistically significant and may explain the reduced leukocyte recruitment observed in the histology.

Adhesion molecules support the extravasation of leukocytes into tissues. In particular, the endothelial adhesion molecules intercellular adhesion molecule 1 (ICAM-1), vascular cell adhesion molecule 1 (VCAM-1), and P-selectin increase during inflammation to support leukocyte extravasation. Given the reduced expression of inflammatory cytokines in the lung of septic CTTN KO mice, we speculated that the expression of adhesion molecules would also be reduced. Indeed, sepsis led to increased expression of ICAM-1, VCAM-1 and P-selectin in WT mice with reduced expression of these adhesion molecules in septic CTTN KO mice comparable to sham controls (Supplementary Figure S1). Overall, cortactin deficiency attenuates the inflammatory response during CLP sepsis.

### 3.3. Genetic Disruption of Cortactin also Ameliorates Systemic Inflammation during Sepsis

**Figure 3:**
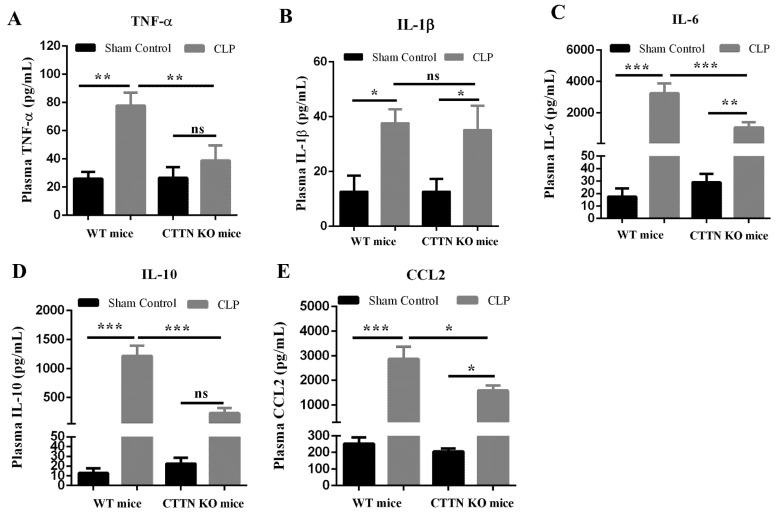
Loss of cortactin ameliorates systemic inflammation during sepsis. Plasma protein levels of the cytokines (**A**) TNF-α, (**B**) IL-1β, (**C**) IL-6, (**D**) IL-10, and (**E**) CCL2 were measured 24 h after CLP sepsis or sham control. Data are represented as mean ± SEM of at least 6 animals per group. * *p* < 0.05, ** *p* < 0.01, *** *p* < 0.001. ns: non-significant.

Sepsis causes systemic inflammation with excessive levels of circulating pro-inflammatory cytokines known as cytokine storm [25]. Thus, the protein levels of inflammatory cytokines in the blood plasma were determined. Plasma TNF-α levels increased significantly in septic mice compared to sham-operated controls, and septic CTTN KO mice showed a significant reduction in TNF-α levels (Figure 3A). By contrast, plasma levels of IL-1β showed a significant increase during CLP sepsis in both WT and CTTN KO mice (Figure 3B). The increase in the levels of IL-6 in septic WT mice was significantly reduced in septic CTTN KO mice (Figure 3C). Additionally, the anti-inflammatory cytokine IL-10 was strongly increased in septic WT mice compared to sham controls; and septic CTTN KO mice showed significantly lower levels of IL-10 compared to septic WT mice (Figure 3D). Finally, the plasma levels of the neutrophil chemoattractant CCL2 were markedly increased in septic WT mice, and there was a significant reduction of plasma CCL2 in septic CTTN KO mice compared to septic WT mice (Figure 3E). Thus, cortactin deficiency also attenuates the systemic inflammatory response during sepsis.

### 3.4. Septic CTTN KO Mice Show Reduced Lung Neutrophil Infiltration during Sepsis

**Figure 4:**
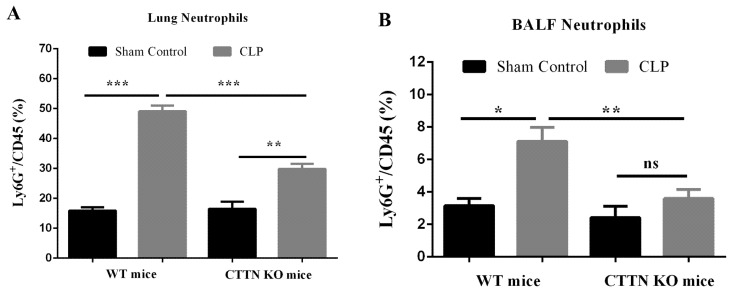
Septic cortactin-deficient mice show reduced neutrophil infiltration into the lung. The percentage of CD45+ LY6G+ neutrophils in lung tissue (**A**) and in BALF (**B**) was determined by flow cytometry 24 h after CLP-sepsis or sham control. Data are represented as mean ± SEM of at least 6 animals per group. * *p* < 0.05, ** *p* < 0.01, *** *p* < 0.001. BALF: Bronchoalveolar lavage fluid.

Septic lung failure is preceded by ALI [25,26], which in turn is characterised by excessive inflammation and neutrophil infiltration [26,27,28]. Given the observed reduced leukocyte presence in the lungs of septic CTTN KO mice, neutrophil numbers in lung tissues were analysed by flow cytometry. CLP sepsis caused massive neutrophil recruitment to the lungs in WT mice (49.1 ± 1.9%), which was significantly reduced in the lungs of septic CTTN KO mice (29.9 ± 1.7%) (Figure 4A). Many infiltrated neutrophils further transmigrate into the bronchoalveolar lumen, where they continue to release proteolytic enzymes and ROS and promote tissue damage [29]. Indeed, a higher frequency of neutrophils in the bronchoalveolar lavage fluid (BALF) of septic WT mice (7.1 ± 0.9%) was found that was significantly lower in the BALF of septic CTTN KO mice (3.6 ± 0.6%) (Figure 4B). These findings show that improved histology and lung injury in septic cortactin-KO mice is likely caused by reduced neutrophil infiltration.

### 3.5. Oxidative Stress Is Ameliorated in the Lungs of Cortactin-Deficient Mice during Sepsis

**Figure 5:**
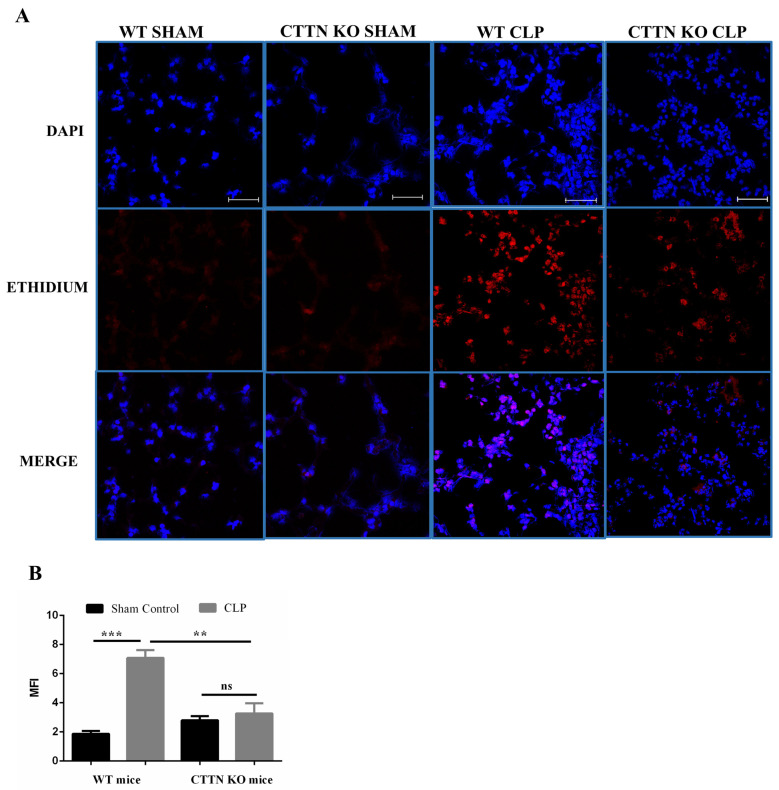
Sepsis-induced oxidative stress is reduced in the lungs of cortactin-deficient mice. (**A**) Representative images from freshly stained lung tissue cross-sections with dihydroethidium (DHE). Pictures were taken with a confocal laser scanning microscope; objective: 40×. Scale bars: 50 μm. (**B**) Quantification of the mean fluorescence intensity (MFI) of oxidised ethidium fluorescence indicating oxidative stress. Data are represented as means ± SEM of at least 4 animals per group. ** *p* < 0.01, *** *p* < 0.001, ns: not significant. DAPI, 4′,6-diamidino-2-phenylindole.

During sepsis, excessive release of ROS leads to oxidative stress that contributes to lung injury [30]. When activated, resident and recruited immune cells are the main producers of ROS [30]. Having observed fewer neutrophils in the lungs of septic CTTN KO mice compared to septic WT mice, the oxidative stress in the lungs during sepsis was analysed. WT and CTTN KO mice subjected to sham surgeries showed no overt oxidative stress (Figure 5A,B). Lungs of septic WT mice showed significantly increased oxidative stress (7.1 ± 0.5). Notably, septic CTTN KO mice had significantly reduced oxidative stress in the lung tissue (3.3 ± 0.7) compared to septic WT mice (Figure 5A,B). Therefore, less neutrophil presence leads to reduced oxidative stress in the lungs of septic CTTN KO mice and less lung tissue damage.

### 3.6. Cortactin Deficiency Prevents Excessive Influx of Neutrophils into the Peritoneum without Affecting Bacteria Count

**Figure 6:**
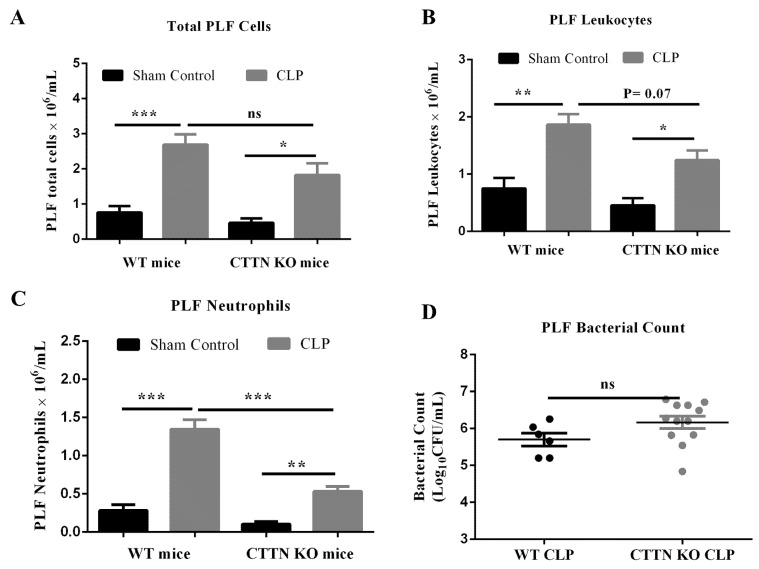
Cortactin deficiency prevents the excessive influx of PMN into the peritoneum without affecting bacteria count. Total numbers of peritoneal cells were counted using a Neubauer chamber (**A**). The numbers of CD45+ total leukocytes (**B**) and CD45+ LY6G+ neutrophils (**C**) in the peritoneal cavity 24 h after CLP sepsis were determined by flow cytometry. (**D**) Bacterial counts were determined by inoculating serially diluted peritoneal lavage fluid (PLF) on tryptic soy agar plate and counting colony-forming units after 24 h incubation at 37 °C; dots represent the number of colony-forming units of PLF from independent mice. Data are represented as mean ± SEM of at least 5 animals per group. * *p* < 0.05, ** *p* < 0.01, *** *p* < 0.001. ns: non-significant.

The outcome of CLP sepsis depends on how efficiently the infection in the peritoneum (primary site of infection during CLP sepsis) can be controlled [31]. Therefore, the levels of recruited neutrophils and bacterial counts in the peritoneum of sham and CLP-operated WT and CTTN KO mice were determined 24 h post-surgery. As expected, CLP induced significant cell influx into the peritoneal cavity of septic WT mice compared to their sham controls. The total number of cells and leukocytes that infiltrated the peritoneum of septic CTTN KO mice was reduced compared to septic WT mice, albeit not in a statistically significant fashion (Figure 6A,B). While CLP sepsis significantly increased neutrophil numbers in the peritoneum of WT mice ((1.4 ± 0.1) × 10^6^ cells/mL), peritoneal neutrophil numbers in septic CTTN KO mice were significantly lower ((0.5 ± 0.1) × 10^6^ cells/mL) compared to septic WT mice (Figure 6C). However, this difference in peritoneal neutrophil levels did not significantly affect the bacterial counts in the respective peritoneum of septic WT (5.7 ± 0.2) vs. CTTN KO mice (6.2 ± 0.2) (Figure 6D). These findings suggest that the number of recruited neutrophils in CTTN KO mice, although reduced, were sufficient to control the peritoneal infection.

### 3.7. Septic Cortactin-Deficient Mice Do Not Develop Neutropenia

**Figure 7:**
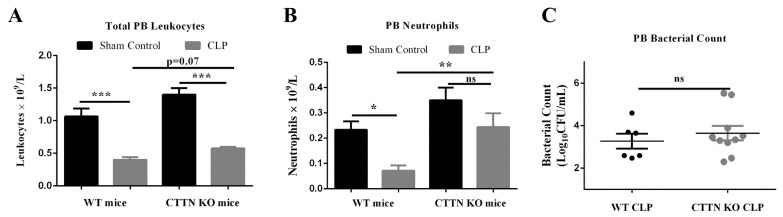
Neutropenia is prevented in septic cortactin-deficient mice. (**A**) Total leukocyte numbers and (**B**) neutrophil numbers in the peripheral blood 24 h after CLP or Sham surgeries in WT and cortactin-KO mice were determined using the automated HemaVet haematology analyser. (**C**) Bacterial CFU in the blood were determined by inoculation of serial dilutions on tryptic soy agar plates and counting CFU after 24 h incubation at 37 °C; dots represent the number of colony-forming units of PLF from independent mice. Data are represented as mean ± SEM. * *p* < 0.05, ** *p* < 0.01, *** *p* < 0.001. PB: Peripheral blood. CFU: colony-forming unit. ns: non-significant.

Major hallmarks of sepsis include leukopenia and neutropenia [26,32,33]. Reduced levels of circulating leukocytes predispose septic patients to opportunistic infections and increase the risk of developing severe sepsis. Given the reduced neutrophil transmigration in CTTN KO mice, we speculated that the peripheral blood of septic CTTN KO mice would have higher leukocyte numbers compared to septic WT mice that are then available to mount a more effective intravascular immune response. CLP sepsis caused decreased levels of total peripheral blood leukocytes in septic WT and CTTN KO mice (Figure 7A). A tendency towards increased total leukocytes in the blood of septic CTTN KO mice ((0.58 ± 0.03) × 10^9^/L) compared to septic WT mice ((0.40 ± 0.04) × 10^9^/L) was observed, albeit not in a statistically significant fashion. CLP sepsis caused neutropenia in septic WT mice 24 h after surgeries ((0.07 ± 0.02) × 10^9^/L). Importantly, neutrophil numbers in the blood of septic CTTN KO mice were significantly higher ((0.24 ± 0.05) × 10^9^/L) compared to septic WT mice, (Figure 7B). Thus, cortactin deficiency prevents neutropenia in septic mice.

The preservation of peripheral blood neutrophils is crucial for the maintenance of an efficient immune response [34]. Interestingly, both septic WT mice and septic CTTN KO mice showed similar bacteria numbers in the blood (Figure 7C). However, higher numbers of neutrophils in the blood of septic CTTN KO mice could imply a better immune response to spreading infection at later stages thus contributing to better survival of septic CTTN KO mice.

Moreover, other haematological parameters during sepsis were analysed in cortactin-deficient mice. Sepsis causes lymphocyte apoptosis resulting in reduced circulating levels of lymphocytes [35,36]. As expected, CLP sepsis induced significant reduction in blood lymphocytes compared to sham-operated groups (Supplementary Figure S2A), but there was no significant difference between lymphocyte counts of septic WT mice (0.30 ± 0.06 × 10^9^/L) compared to septic CTTN KO mice (0.28 ± 0.04 × 10^9^/L). Additionally, sepsis causes a reduction in blood platelet counts [37]. Platelets interact with neutrophils and promote their extravasation during inflammation [38]. Platelets have also been shown to combat lung injury by increasing the levels of transforming growth factor β (TGF-β), and attenuating the levels of TNF-α and neutrophil elastase in blood and lungs during systemic inflammation [39]. However, no significant changes in platelet numbers in the blood of either septic WT (645.6 ± 55.4 × 10^9^/L) or CTTN KO mice (546 ± 134.9 × 10^9^/L) were observed compared to sham-operated mice (Supplementary Figure S2B). Additionally, there were no significant differences between sham and CLP-operated WT and CTTN KO mice in other haematological parameters such as erythrocyte count, haemoglobin levels, haematocrit, and total solids (Supplementary Figure S2C–F). Thus, except for neutrophil numbers, cortactin deficiency did not alter haematological parameters during sepsis.

### 3.8. Delayed Neutrophil Depletion Provides No Additional Protective Effect in CTTN KO Mice

At the onset of sepsis, neutrophils are required for controlling bacterial infection. However, neutrophils become dysregulated later during sepsis, leading to delayed apoptosis and accumulation in organs [40]. Excessive neutrophil levels in organs during sepsis cause tissue injury. These Janus-like roles of neutrophils during sepsis are further highlighted by the fact that neutrophil depletion before sepsis onset results in higher bacteraemia and mortality compared to later depletion 12 h after sepsis onset that improves survival [10]. To determine whether the diminished levels of neutrophils in the lungs are the reason for the observed protective effect in septic CTTN KO mice, neutrophil depletion experiments were performed. Neutrophils were depleted from peripheral blood 12 h after CLP surgery by intravenous injection of anti-mouse Gr-1 antibody or isotype control antibody as previously described [10]. Intravenous administration of the anti-mouse Gr-1 antibody depleted 99% of circulating neutrophils (Supplementary Figure S3A,B). Importantly, neutrophils in the blood of both septic WT (1.64 ± 0.3%) and CTTN KO mice (7.87 ± 3.3%) were similarly and significantly reduced upon injection of the anti-mouse Gr-1 antibody compared to isotype-injected controls (Supplementary Figure S3C). Septic WT mice that received isotype control antibody had higher numbers of peritoneal neutrophils (2.03 ± 0.5 × 10^6^ cells/mL) compared to septic CTTN KO mice (0.78 ± 0.3 × 10^6^ cells/mL) injected with the isotype control antibody. The peritoneal neutrophil levels were strongly reduced after neutrophil depletion in both septic WT (0.26 ± 0.1 × 10^6^ cells/mL) and septic CTTN KO mice (0.14 ± 0.1 × 10^6^ cells/mL), but there was no more statistically significant difference between the septic WT and CTTN KO groups (Supplementary Figure S3D).

**Figure 8:**
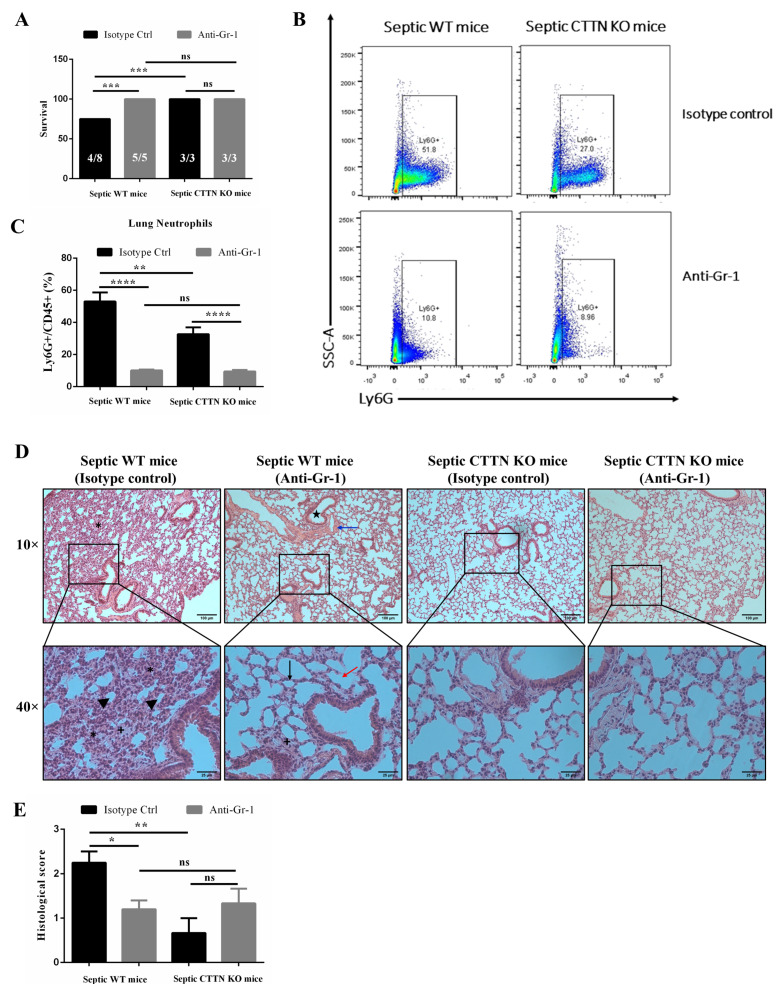
Neutrophil depletion had no additional effect on septic cortactin-deficient mice. (**A**) Survival analysis 24 h after CLP surgery and 12 h after injection of the respective antibodies. (**B**) Representative flow cytometry plots showing similar neutrophil levels in the lungs when injected with either anti-mouse Gr-1 antibody or isotype control antibody 12 h after CLP sepsis. Flow cytometry of lung tissue suspensions was done 24 h after the onset of sepsis (12 h after injection of the antibodies). (**C**) Frequency of CD45+ Ly6G+ neutrophils in the lungs of septic mice 24 h after CLP surgery. (**D**) Representative haematoxylin–eosin-stained images of septic WT mice and septic CTTN KO mice injected with either anti-mouse Gr-1 antibody or isotype control antibody. Septic WT mice that received the isotype control antibody showed major signs of lung inflammation and injury as evidenced by excessive leukocyte infiltration (+), alveolar wall thickening (asterisks), and alveolar space closing and collapse (black arrowheads). Neutrophil-depleted septic WT mice showed mild lung inflammation and injury with minimal leukocyte influx, reduced alveolar wall thickening, and a better preserved alveolar (red arrow) and bronchiolar areas (star), and normal blood vessel (blue arrow). Both septic CTTN KO mice that received isotype control antibody or were neutrophil-depleted showed well-preserved lung histology. (**E**) Histological scoring of inflammatory changes in the lungs. 0: absence of inflammation, 1: low inflammation, 2: moderate inflammation, and 3: high inflammation. Data are represented as means ± SEM of at least 3 animals per group. * *p* < 0.05, ** *p* < 0.01, *** *p* < 0.001, **** *p* < 0.0001, ns: non-significant.

Next, the effect of delayed neutrophil depletion on sepsis survival and lung histology was analysed. Septic WT mice depleted of neutrophils 12 h after onset of sepsis had improved survival 24 h after sepsis compared to septic WT mice injected with the isotype control antibody (Figure 8A). The survival of 50% in WT mice injected with isotype control antibody recapitulates our previous observation with non-injected WT CLP mice (Figure 1A). Interestingly, no difference in survival between neutrophil-depleted WT mice, control CTTN KO mice, and neutrophil-depleted CTTN KO mice was observed, with all mice surviving. Analysing neutrophil infiltration into the lung, significantly reduced neutrophil levels in the lungs of both septic neutrophil-depleted WT (10.12 ± 0.5%) and CTTN KO mice (9.43 ± 1.0%) were observed compared to septic mice that received isotype control antibody (Figure 8B,C). Importantly, there was no significant difference between lung neutrophils in neutrophil-depleted septic WT mice and neutrophil-depleted septic CTTN KO mice (Figure 8B,C).

Histopathological analysis revealed that septic WT mice injected with isotype control antibody showed significant inflammatory changes in lung histology samples, indicating lung damage as seen by massive leukocyte infiltration, alveolar wall thickening, and alveoli collapse (Figure 8D,E). Consistent with better survival in neutrophil-depleted septic WT mice, lung tissue histomorphology was better preserved with less leukocyte influx, reduced alveoli collapse, and alveolar wall thickening (Figure 8D,E). Depleting neutrophils in septic CTTN KO mice had no additional protective effect on lung histology, which appeared almost normal in both isotope-injected and Gr1-injected mice. Neutrophil-depleted septic WT mice showed a significantly lower histological score of 1.2 ± 0.2 compared to the score of 2.3 ± 0.3 in WT mice that received the isotype control. Importantly, there was no significant difference between the histological score of neutrophil-depleted septic WT mice and neutrophil-depleted septic CTTN KO mice (1.2 ± 0.2 vs. 1.3 ± 0.3, respectively). Therefore, we conclude that the excessive influx of neutrophils into the lungs after 12 h of sepsis drives lung injury and promotes mortality during murine sepsis, and that cortactin deficiency prevents this late excessive neutrophil recruitment into the lung leading to preserved lung functions and improved survival.

## 4. Discussion

Despite many efforts on finding treatments for sepsis, there is still no FDA-approved drug available for its management [41]. Thus, research providing better understanding of the mechanisms driving sepsis pathogenesis is warranted to facilitate the search for new therapeutic targets. In this study, we investigated whether the ABP cortactin, known to support neutrophil extravasation across the endothelial barrier, plays an important role in sepsis pathogenesis. Using a mouse model of CLP sepsis, we showed that cortactin deficiency improved survival in septic mice by attenuating the inflammatory response and reducing neutrophil infiltration that together prevented lung tissue damage.

ALI is a key feature of sepsis and characterised by exacerbated inflammation and excessive influx of neutrophils into the lung [24,26]. ALI is also one of the triggers of mortality in sepsis. The deficiency of cortactin improved histopathological changes occurring in the lung tissue during sepsis and, in line with our previous observation that cortactin-deficient mice have defects in neutrophil recruitment, we observed little neutrophil infiltration into septic lungs. It is interesting as to why the absence of cortactin, an ABP required for the maintenance of the endothelial barrier via the activation of the small GTPase Rap1, did not exacerbate lung pathology during sepsis. Notably, cortactin-KO mice showed no developmental lethality or any obvious phenotype and had a normal lifespan [16]. It is currently unknown whether another ABP compensates for the loss of cortactin to maintain the endothelial barrier in the lung; and this will be an important topic for future studies. For example, the ABP vasodilator-stimulated phosphoprotein (VASP) could compensate for the loss of cortactin and stabilise the endothelial barrier in the lung because VASP is known for its barrier-stabilising properties in the lung [42,43].

The cytokine storm, a key feature of sepsis that has been linked to increased risk of mortality, is characterised by a life-threatening systemic inflammation caused by high circulating levels of the proinflammatory cytokines TNF-α, IL-6, and IL-1β [44,45,46]. Interventions aimed at reducing the cytokine storm have shown improvements in sepsis outcome [47,48,49]. In our study, we show that the cytokine storm was significantly attenuated in cortactin-deficient mice during polymicrobial CLP sepsis. During inflammation and sepsis, the transcription factor nuclear factor κB (NF-κB) is activated [50,51], which drives excessive inflammatory cytokine expression [52]. NF-κB is also important for the recruitment of neutrophils since NF-κB activation promotes the expression of chemokines, ICAM 1, and other endothelial adhesion molecules [53,54,55]. Considering reduced cytokine and adhesion molecule expression in the absence of cortactin, it is tempting to speculate that improved survival in septic cortactin-deficient mice may be linked to diminished NF-κB activation. However, this idea needs to be verified in future studies to elucidate the exact mechanisms by which cortactin regulates the production of cytokines and chemokines.

Endothelial activation by cytokines, pathogen-associated molecular patterns, and damage-associated molecular patterns during sepsis causes increased expression of cell adhesion molecules, thus promoting transmigration of leukocytes. Neutrophil extravasation across the endothelial barrier is an active process, which requires adhesive interactions between the vascular endothelium and the neutrophil. Genetic ablation of the adhesion molecule ICAM-1 improved survival in murine sepsis by attenuating neutrophil infiltration into the lungs and preventing lung injury [56]. Cortactin is important for appropriate ICAM-1 clustering and functions during neutrophil extravasation [16]. Cortactin supports ICAM-1 clustering during slow rolling of neutrophils and arrest [16]. Therefore, our data showing decreased neutrophil levels and oxidative stress in the lung could be a consequence of both reduced ICAM-1 de novo production and clustering [16]. Whether ICAM-1 clustering is also reduced in the context of cortactin-deficient septic lungs should be investigated in the future. Moreover, the chemokine CXCL-1 is an important neutrophil chemoattractant during inflammation in general [57,58] and sepsis in particular [59]. Furthermore, the levels of the chemokine CCL2 are increased in severe septic patients and correlate with organ failure and mortality [60,61], and CCR2 (the receptor for CCL2) expression on neutrophils is induced during sepsis, thus triggering neutrophil recruitment to peripheral organs such as the lung and kidney that express large amounts of CCL2 [62,63]. Sepsis-induced neutrophil infiltration and lung damage was attenuated upon blocking CCL2 [64]. Therefore, the reduced lung inflammation and neutrophil influx could be explained by the lower levels of CXCL-1 and CCL2 observed here.

Neutrophils are important in the development of sepsis-induced lung damage [11,26,29]. While infiltration of the lungs with few sentinel neutrophils is necessary for immune surveillance that occurs without causing any injury to the lung tissue, neutrophils become hyperactivated and dysregulated during sepsis, and their massive infiltration of the lung is accompanied by tissue damage [29,40]. These neutrophils stay longer in the lung and release more destructive molecules such as proteolytic enzymes and ROS that exacerbate tissue injury [65,66,67]. Evidence implicates neutrophils as major mediators of lung injury during sepsis. For example, lung autopsy specimens from patients with multi-organ failure showed massive infiltration with neutrophils [13]. Additionally, leukodepletion in patients with systemic inflammatory response syndrome led to improved respiratory functions [68]. Experimentally, excessive neutrophil infiltration into tissues such as the lung has been shown to precede organ failure [69], and interventions aimed at depleting neutrophils ameliorated organ dysfunction in mice, although in a strictly time-dependent manner [10]. Accordingly, we confirmed that CLP sepsis caused massive neutrophil infiltration into lungs and bronchoalveolar space in septic WT mice, a phenotype that was significantly reduced in septic cortactin-deficient mice. Neutrophils produce and release ROS needed to fight invading pathogens. However, in excess, ROS also contribute to tissue damage [70], so that during sepsis the natural antioxidant defences are rapidly depleted, leading to severe oxidative stress [30]. It is, therefore, likely that the increased oxidative stress in the lungs of septic WT mice observed here is a consequence of higher neutrophil numbers in the lung, and vice versa the reduced oxidative stress in the lungs of septic CTTN KO mice is the result of fewer neutrophil presence leading to better preserved tissue histomorphology and significantly reduced lung damage. In this context, we recently showed that the absence of HS1, a cortactin homolog exclusively expressed in haematopoietic cells, also improved survival during murine sepsis by attenuating excessive neutrophil recruitment into the lungs and reducing neutrophil-inflicted lung damage [71], thus independently proving that proper control of neutrophil recruitment to the lungs is critical for the outcome of sepsis.

The early infiltration of the peritoneum with leukocytes including neutrophils is important in limiting the infection thus ensuring better sepsis outcome [68]. By contrast, higher numbers of recruited neutrophils in the peritoneum following CLP sepsis are linked to poor control of bacteria due to defective phagocytosis and bacterial killing ability 24 h after the onset of murine sepsis [32,72]. Increasing evidence suggests that continuous hyper-stimulation of cells with contradictory stimuli such as pro- and anti-inflammatory cytokines, which occurs in sepsis, transforms recruited cells into a zombie-like phenotype characterised by impaired effector functions [72,73]. Thus, the higher levels of pro- and anti-inflammatory cytokines in septic WT mice possibly render neutrophils less effective in controlling the bacterial infection compared to septic CTTN KO mice with lower cytokine levels, i.e., neutrophils in CTTN KO mice may not develop into non-functional zombies. Hence, the fewer peritoneal neutrophils in septic CTTN KO mice may still be sufficient to fight bacteria, thus explaining the similar bacteria burden in the peritoneum of septic WT and CTTN KO mice. CLP sepsis also causes translocation of bacteria from the peritoneum into the circulation. The findings of similar bacteria count in the blood of septic WT and septic CTTN KO mice are consistent with similar bacteria counts in the peritoneum and could mean that both mice had similar levels of bacterial translocation into the blood. Thus, differences in peripheral blood bacteria cannot explain the different sepsis outcome in WT and CTTN KO mice.

Neutrophils are the first cells of the innate immune system that are activated and recruited during sepsis [59], and the excessive movement of immune cells including neutrophils into tissues causes leukopenia and neutropenia [27,33,34], conditions that predispose sepsis patients to nosocomial infections. In this context, it is important to note that cortactin deficiency did not affect haematological parameters and blood cell numbers except for neutrophils, as we have previously reported [16], and confirmed in this study. Our observation that neutropenia is prevented in septic cortactin-KO mice is likely the consequence of defective neutrophil transmigration and ensures an effective intravascular immune response to better control systemic infection. Together, it is reasonable to conclude that the protective effect of cortactin deficiency during sepsis is not linked to haematological differences other than the differences in neutrophil recruitment patterns. However, although cortactin is not expressed in neutrophils, it is expressed in other cells such as tissue-resident macrophages and epithelial cells. Therefore, we cannot entirely rule out indirect effects resulting from cortactin deficiency in these cells. However, the conclusion that most of the protective effect stems from endothelial cortactin regulating neutrophil extravasation is further supported by the data of our neutrophil depletion experiment. While neutrophil depletion before the onset of sepsis is detrimental, as mice easily succumb to the infection and develop organ injury, delayed depletion (12 h after CLP sepsis) leads to better clearance of bacteria and attenuated organ injury [10]. This previous study thus showed the critical role of early neutrophil presence at the focus of infection for a positive outcome, while simultaneously proving that later excessive infiltration of organs with neutrophils triggers organ damage [10]. Our data are in line with these findings and confirm that upon depleting neutrophils 12 h after CLP surgery, neutrophil numbers in the lung and lung tissue damage are reduced. Depletion removes up to 99% of circulating neutrophils (Supplementary Figure S3). Therefore, early depletion at the onset of CLP inhibits neutrophil recruitment almost completely because they are simply not available, in other words, no neutrophils reach the site of infection to control it, thus explaining the worse survival. By contrast, cortactin deficiency only reduces and does not ablate neutrophil recruitment, so that neutrophils in certain amounts are recruited even in the absence of cortactin, which can contribute to fighting the infection. Therefore, cortactin deficiency does not exactly phenocopy complete neutrophil depletion during CLP. On the other, when neutrophils are depleted 12 h after CLP, many neutrophils have reached the site of infection during the first 12 h that can fight invading bacteria. Later after 12 h excessive recruitment is then completely prevented by the depletion so that excessive neutrophil recruitment that would contribute to organ damage rather than pathogen fighting is prevented, and this is a scenario that is rather phenocopied by cortactin deficiency. In other words, our finding that cortactin deficiency did not provide any additional protective effect on top of neutrophil depletion proves that the protective effects of cortactin deficiency during sepsis are owing to its inhibition of neutrophil recruitment. 

## 5. Conclusions

We conclude that reduced pulmonary neutrophil influx in amounts sufficient to control the infection but insufficient to cause tissue damage during sepsis constitutes the underlying mechanism explaining the improved survival of mice lacking cortactin.

In summary, our study shows that cortactin deficiency is protective during murine sepsis by ameliorating the exaggerated inflammatory response and preventing neutrophil-inflicted tissue damage. Thus, interfering with cortactin-supported neutrophil extravasation could be a promising pharmacological strategy for the treatment of sepsis.

## Data Availability

Not applicable.

## Supplementary Materials

The following supporting information can be downloaded at: https://www.mdpi.com/article/10.3390/biomedicines10051019/s1, Figure S1: Loss of cortactin ameliorates the gene expression of adhesion molecules in the lung during sepsis; Figure S2: Haematological analysis after CLP sepsis; Figure S3: PMN depletion efficiency in wild-type and cortactin-deficient mice subjected to CLP sepsis.
