# Supplementary material for: The Actin-Binding Protein Cortactin Promotes Sepsis Severity by Supporting Excessive Neutrophil Infiltration into the Lung
Source: Biomedicines. 2022 Apr 28;10(5):1019. doi: 10.3390/biomedicines10051019 (PMC9139066; doi:10.3390/biomedicines10051019)
Supplement: Supplementary file 1 [file biomedicines-10-01019-s001.zip › biomedicines-1651080-supplementary.pdf]

Supplementary data

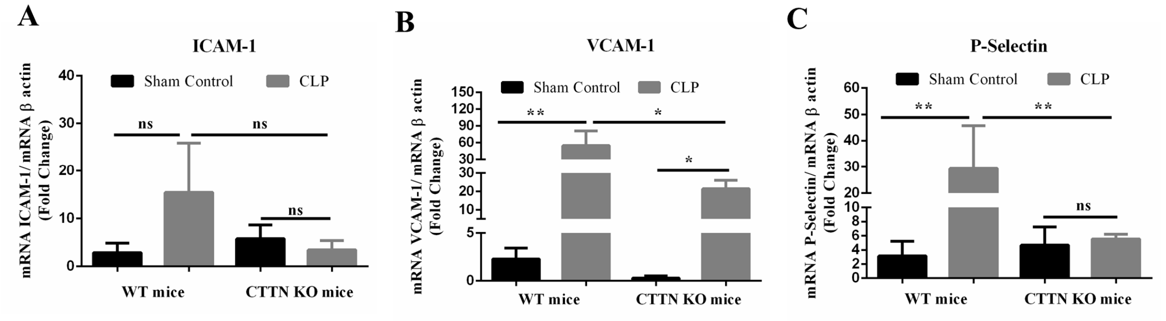

Figure S1: Loss of cortactin ameliorates the expression of adhesion molecules in the lung during sepsis. Relative gene expression of (A) ICAM-1, (B) VCAM-1, and (C) P-selectin in the lung tissues of WT and CTTN KO mice 24 h after CLP/Sham surgeries. The ΔΔCt method was used to calculate gene expression, and results are shown as fold change normalized to the housekeeping gene β-actin. Data are represented as means ± standard error of the mean of at least 5 animals per group. *$p < 0.05$, **$p < 0.01$, ns: non-significant.

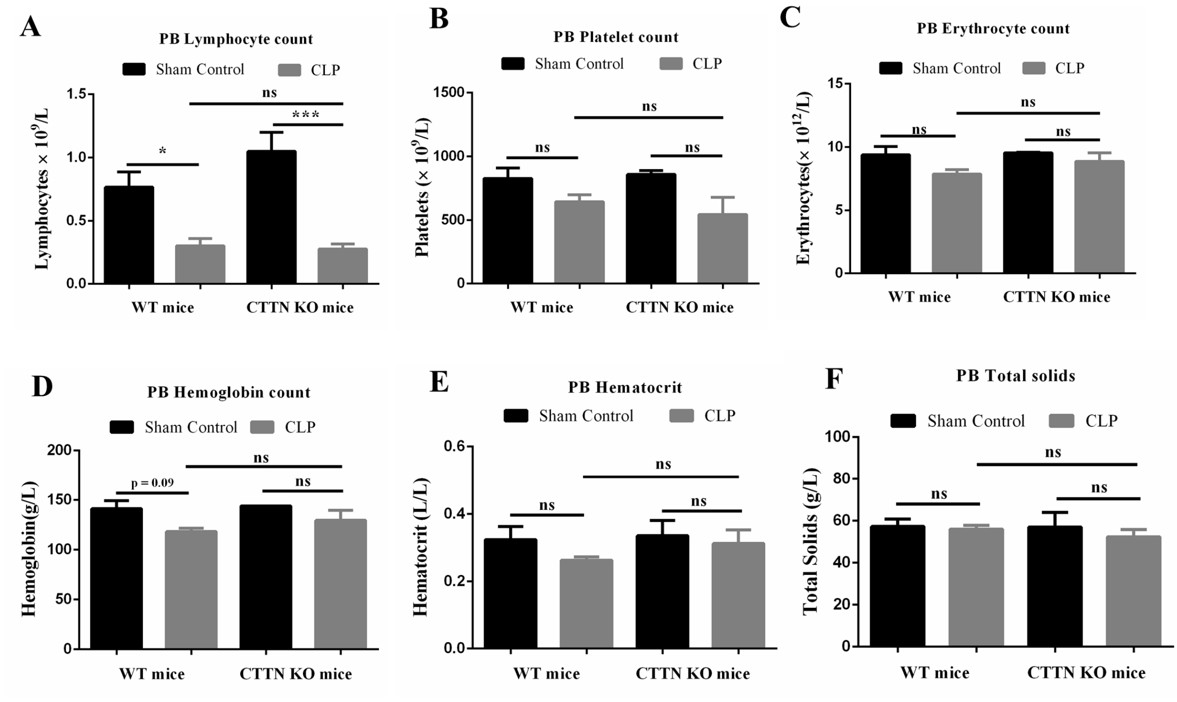

Figure S2: Haematological analysis after CLP sepsis. Peripheral blood counts of (A) Lymphocytes (B) Platelets, (C) Erythrocytes, (D) Haemoglobin, (E) Haematocrit, and (F) Total solids determined 24 h after CLP or Sham surgeries in WT and CTTN-KO mice using the automated HemaVet hematology analyzer. Data are represented as means ± standard error

of the mean of at least 5 animals per group. *p < 0.05, ***p < 0.001. PB: Peripheral blood. ns: non-significant.

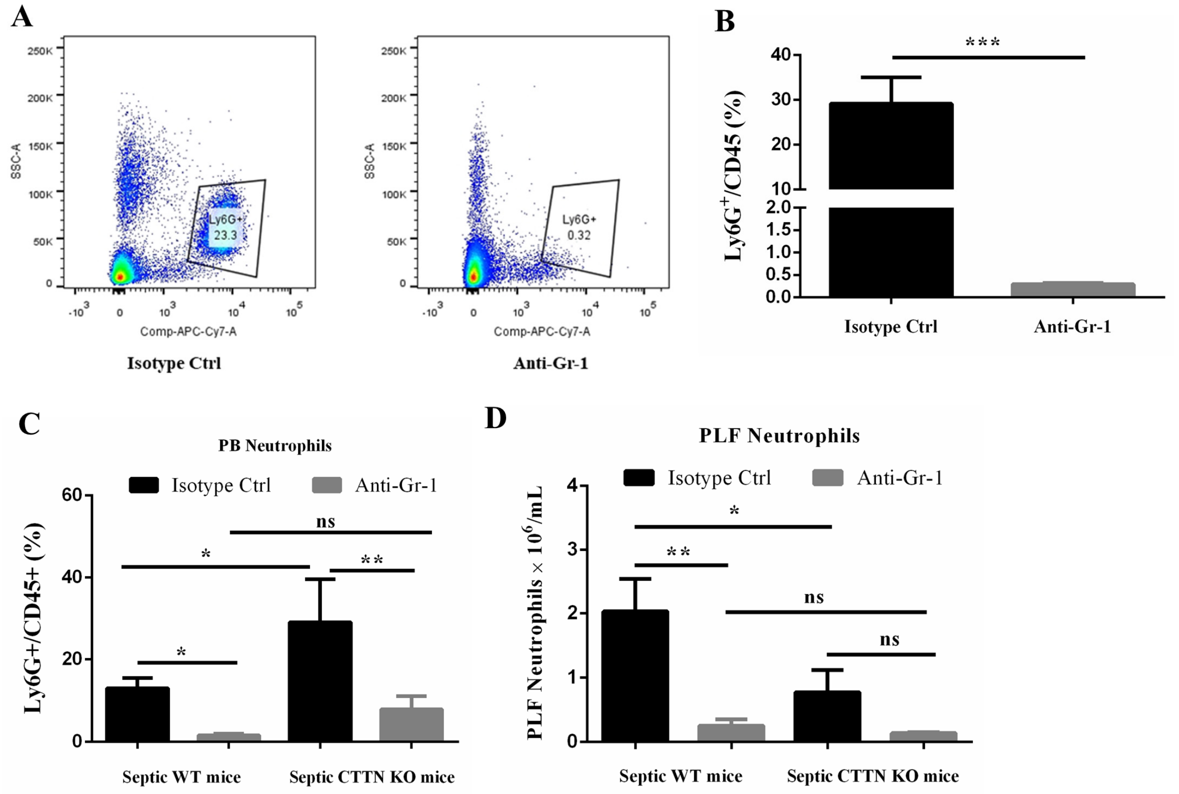

Figure S3: Neutrophil depletion efficiency in wild-type and cortactin-deficient mice subjected to CLP sepsis. (A) Representative flow cytometry plots showing efficient depletion of neutrophils in WT mice using anti-mouse Gr-1 antibody (B) Quantification of the plots in (A) showing $CD45^+Ly6G^+$ neutrophil frequency in peripheral blood. $CD45^+Ly6G^+$ neutrophils in isotype-injected and neutrophil-depleted septic WT and CTTN KO mice in peripheral blood (C), and PLF (D) 24 h after CLP sepsis. Data are represented as means ± standard error of the mean of at least 3 animals per group. *p < 0.05, **p < 0.01, ***p < 0.001, ns: non-significant. PB: Peripheral blood. ns: non-significant. PLF: Peritoneal lavage fluid.
